# A Qualitative Study of Perceived Risk of Occupational Exposure to HIV and Use of Post Exposure Prophylaxis Services Among Health-Care Workers in Tanzania

**DOI:** 10.24248/EAHRJ-D-19-00018

**Published:** 2019-11-29

**Authors:** Edith AM Tarimo, Kijakazi O Mashoto

**Affiliations:** a Muhimbili University of Health and Allied Sciences, Dar es Salaam, Tanzania; b National Institute for Medical Research, Dar es Salaam, Tanzania

## Abstract

**Background:**

Occupational exposure to HIV continues to present a risk of HIV infections to health-care workers (HCWs) in low-income countries. Since 2005, policies in Tanzania have been in place to guide the implementation of HIV/AIDS post-exposure prophylaxis (PEP) interventions in the workplace. However, little is known about how frontline HCWs experience and view these interventions. This study aimed to explore how HCWs perceive their HIV infection risk and capture their experiences of workplace HIV/AIDS interventions.

**Methods:**

A descriptive qualitative design was used. Medical and nonmedical personnel from 2 hospitals in the Pwani and Dodoma regions of Tanzania participated in the study. We conducted 22 In-depth interviews (IDIs) with HCWs (heads of departments, hospital units, or sections). A content analysis approach was used.

**Results:**

The HCWs perceived and reasoned that working in medical wards, incinerator units, dental units, obstetric wards, laundries, laboratories, and mortuaries exposed them to HIV acquisition risk. Many of the medically trained personnel reported that invasive procedures exposed them to some risk of HIV infection. Nonmedical personnel reported to be potentially exposed to HIV infection while incorrectly handling discarded needles and blades (sharps). Although most HCWs expressed awareness about the availability of postexposure prophylaxis (PEP), not all HCWs knew where to report and whom to contact in case of accidents. Ignorance about the implications of exposure to contaminated sharps hindered PEP use among certain cadres. Also, some PEP users were reported to experience side effects, but they were motivated to complete the doses to remain healthy.

**Conclusion:**

Occupational exposure to HIV infection remains a significant concern to HCWs, particularly among nonmedical cadres. Despite expressed awareness about infection prevention and control, the reporting channels and the strategies to promote recognition of the importance of using PEP services after exposure need to be strengthened.

## INTRODUCTION

In 2018, 37.9 million people globally were living with HIV, of which 1.7 million became newly infected with HIV, and sub-Saharan Africa remains the most affected region.^1^ Tanzania is among the sub-Saharan Africa countries affected by the HIV epidemic. On average, 4.6% of adults between 15 and 49 years of age were infected with HIV in Tanzania.^1^ Occupational exposure poses a risk of HIV transmission and may increase work-related diseases risk. In the context of hospital settings, the most common exposures are needle-stick injuries and splashes with blood and body fluids.^2^ Health-care workers (HCWs) are at risk of occupational acquisition of HIV infection, primarily due to accidental exposure to infected blood and bodily fluids.^3–4^ The use of personal protective equipment (PPE), adherence to universal precautions, effective post-exposure management, engineered safer devices, injury surveillance, and relevant legislations are amongst strategies which are designed to maximise the safety of HCWs and patients in health-care setting.^5^

The level of risk depends on the HIV prevalence of patients and the precautions the HCWs observe during a surgical, medical and clinical procedures while dealing with blood and bodily fluids.^6^ Prior to the introduction of antiretroviral therapy (ART), the occurrence of HIV transmission varied according to the type of exposure and skin condition of the HCW. For example, in a review of prospective studies of seroconversion after occupational exposure to an HIV infected source, 20 of 6,135 cases, (0.33%) became infected following percutaneous exposure; 1 case out of 1,143 (0.09%) was infected following exposures on the mucosa of HCW; and there were no cases after 2,712 intact skin exposures.^7^ In a multisite case-control study by Centre for Disease Control (CDC), needle stick injuries from an infected source revealed that the depth of injury, needle placement in a vein or artery increased the risk of acquiring HIV. In this situation, most cases appeared to be injured by a hollow bore as opposed to a solid needle.^8^ Different cadre of HCW are also at different risks of exposure; nurses appeared to have reported the most frequent blood and body fluids exposure followed by resident physician and allied professionals (HCWs who are not physicians, dentists or nurses).^9^ Fatigue associated with long working hours and sleep deprivation among medical trainees have been reported to increase the risk of needle stick injuries.^10^

In low-income countries, the risk of HIV infection due to occupational exposure is increased by a range of factors including but not limited to high workload, inadequate or unavailability of protective gears, and lack of knowledge on standard precaution.^11–12^ Whilst studies indicate that the estimated risk of occupational HIV transmission in health-care settings is low.^3,13^ However, the risks of exposure and subsequent sero-conversion is high in high prevalence and resource limited settings especially if PEP strategies are inadequate.^3^

Studies have indicated that the perception of risk of occupational HIV transmission amongst HCWs is high.^14–16^ However uptake and availability of postexposure prophylaxis (PEP) does not match the occurrence of exposure.^12^ PEP is a medical response to prevent transmission of pathogens after potential exposure and refers to comprehensive management instituted to minimise the risk of infection following potential exposure to HIV. It includes first aid, counseling, risk assessment, relevant laboratory investigations based on the informed consent of the exposed person and source and depending on the risk assessment, the provision of short term of antiretroviral drugs, along with follow-up evaluation.^17–19^ Post Exposure Prophylaxis for HIV exposure is best when started within 2 hours, with little benefit after 72 hours. The prophylaxis needs to be continued for 28 days. HCWs may be aware of the exposure to HIV risks, but they may lack understanding of logistics involved, PEP usage, and outcome in HIV care settings. With this in mind, there is a need for the understanding of perceived risk of occupational HIV transmission, practice, and factors influencing the use of PEP among Tanzanian HCWs Therefore, this study aimed to explore HCWs’ perceptions and experiences on health safety, availability, and use of PEP in particular. In this study, an HCWs were medical or nonmedical personnel working in hospitals in 2 regions of Tanzania.

## METHODS

### Study Design and Sampling

This is a descriptive qualitative design. Purposive sampling was used to recruit HCWs from 2 hospitals, namely, Tumbi in Pwani Region and Dodoma in Dodoma Region. According to the 2012 national census, the Pwani and Dodoma regions had a population of 1,098,668 and 2,083,588, respectively.^20^ According to the Tanzania HIV Indicator Survey, the HIV prevalence for Pwani and Dodoma regions was 5.5% and 5%, respectively.^21^ Tumbi and Dodoma hospitals were purposefully selected. Tumbi Hospital was selected because of its popularity of frequently attending clients involved in car road accidents along highway roads to Morogoro region, Segera, Moshi and Arusha, and Dar es Salaam,^22^ leading to increased risk of exposure to HIV among HCWs when attending injured clients. The Dodoma Hospital, being in the central region in Tanzania, was purposefully selected to represent other regions in the country which experienced regular or few incidents of road accidents.^13^ Both hospitals have got well established Care and Treatment Clinics to provide HIV/AIDS services. Dodoma is a government-owned hospital with a bed capacity of 420 and serves 118,000 patients per year as a regional referral hospital. Tumbi is a parastatal owned hospital with 253 beds and serves as a designated regional referral hospital to 300,000 patients per year. Both hospitals are located in urban areas and provide inpatient and outpatient services such as general OPD, care, and treatment clinic (CTC), voluntary counseling and testing (VCT), reproductive and child health (RCH) and clinics for Tuberculosis and Leprosy, Diabetes, eye, dental, gynecology, and obstetrics.^13^

### Data Collection

An in-depth interview (IDIs) guide was developed and pre-tested amongst HCWs in a different hospital in Dar es Salaam. Topics on the IDI included perceived risk of a HCW acquiring HIV infection at work place, available services for those exposed to HIV infection, awareness about Post Exposure Prophylaxis, reporting channels after HIV exposure, perceived risky sections at workplace, and recommendations to improve HIV prevention at workplace. Trained research assistants conducted all interviews in privacy at the workplace, using an interview guide. The main questions in the guide were followed by a probing set of questions according to the responses. IDIs with the HCWs (Table 1) were audio-recorded and lasted between 20 and 30 minutes. The information collected was based on the principles of theoretical saturation.^24^ Data were collected between February and March 2012.

**TABLE 1.** Health-Care Workers

SN	Unit	Number of Participants
1	Deputy In-charge Out Patient Department	1
2	Laboratory Deputy In-charge	2
3	Mortuary	2
4	Theatre In-charge	1
5	Laundry In-charge	2
6	Voluntary Counseling and Testing/Nurse RCH	2
7	Surgical ward	1
8	Medical Ward In-charge	2
9	Labor/Obstetric ward	2
10	Incinerator/Waste management	1
11	Care and Treatment Clinic (CTC)/PEP personnel	2
12	Dental Offcer In-charge	2 1
13	Health Secretary	1
14	Nurse Assistant In-charge	1
	**Total**	**22**

### Data Analysis

Content analysis approach was loosely guided by Kvale.^24^ A research assistant transcribed the audio-records verbatim. The authors coded all transcripts on the margin of each transcript. The codes were sorted manually into categories. Final theme and categories were discussed between the 2 authors; differences were negotiated for consensus. Quotes were used to illustrate participants’ views are reflected in the paper. Hospitals were assigned codes which were used to distinguish the 2 regions whilst still ensuring anonymity, but also to protect the participants’ identity.

### Ethical Considerations

Ethical clearance was obtained from the Medical Research Coordinating Committee (MRCC) of the National Institute for Medical Research (NIMR) Secretariat (Re.NIMR/HQ/R.8a/Vol. IX/1278). Regional and District/Municipal Government and Health Authorities for Pwani and Dodoma Regions also permitted for this study to be undertaken in the selected Hospitals. The final decision to participate in the study was taken by the HCWs after reading the consent form. The HCWs voluntarily consented to take part in the study after reading and understanding the consent form. The consent form comprised of matters relating to confidentiality, privacy, risks, and freedom to withdraw from the study at any time. The consent to participate was audio-recorded. To maintain the confidentiality of the HCWs, names, or individual identities are not reported in this paper.

## RESULTS

### Description of the Participants

Twenty-two HCWs from the following unitsout Patient Department, Laboratory, Mortuary, Operating Theatre, Laundry, Reproductive Child Health (RCH), Surgical ward, Medical ward, Labor/Obstetric Ward, Waste management, Care and Treatment Clinic (CTCs), and Dental Clinic were interviewed (Table 1). The Outpatient In-charge, Theatre In-charge, Surgical Ward In-charge, Waste Management Person, Health Secretary, and Nurse Assistant In-charge in one of the hospitals were not available during data collection.

### Theme and Categories

Under the central theme of ‘Perceived risks of acquiring HIV infection and experiences of workplace HIV/AIDS interventions’, 3 categories emerged (Table 2). The first category describes how HCWs perceived the workplace as a source of risks of HIV transmission. The second category underscores the reported availability and access to Post Exposure Prophylaxis (PEP) at the workplace. The third category addresses the facilitators of using PEP after encountering risks of HIV transmission.

**TABLE 2:** Theme and Categories

Theme	Categories
Perceived risks of acquiring HIV infection and experiences of workplace HIV interventions	Perceived sources of risks of HIV transmission at the workplace
	Availability and access to Post Exposure Prophylaxis Motivations and diffculties of using Post Exposure Prophylaxis

### Perceived Sources of Risk of HIV Transmission at the Workplace

The HCWs stated that they came across various situations that could expose them to HIV transmission. They perceived some situations riskier than others. For example, medical wards were considered as bearing a greater risk of transmission as compared to other wards. HCWs felt that because some patients were HIV infected, and the HCWs were likely to get into contact with HIV infected blood or contaminated instruments during provision of care. One HCW stated:

*Unfortunately, you may be attending a patient with AIDS… perhaps during intravenous fluid insertion; you may punch yourself either accidentally or because of carelessness. Also, a used needle may accidentally drop on you. Such possibilities exist* (Medical In-charge, Region 2)

Other HCWs emphasised that the risk of infection in medical wards is wide spread, particularly if HCWs do not use protective gear such as gloves and safety glasses. They perceived needle pricks, blood splashes to eyes, nose, mouth, and other parts of the body as common risk factors for occupational HIV transmission in the medical wards. Thus in their views, any procedure that involves blood can cause HIV transmission if the HCWs’ skin is not intact. Also, they said that the risks of HIV exposure in the medical wards were exacerbated by improper disposal of wastes. The personnel from the waste management unit complained that they were affected because of repeated experiences of handling improperly disposed sharps. They complained that the disposal bags were misused. One HCW said:

*He/she [medical ward personnel] may put a syringe in the blue instead of a red bag. When the waste management personnel come to collect wastes, he/she may not think of coming in contact with dangerous materials in the blue bag… many times they end up with cuts and pricks… if that person is not aware of the reporting channels, he/she will not act; he/she will think that it is just a small issue…he/she may end up with HIV infection* (Waste management, Region 2)

Also, HCWs from incinerator unit were concerned about improper waste disposal and lack of adequate knowledge on what constitutes and what to do in the event of a high risk exposure. One HCW elaborated:

*The wastes are supposed to be packed in different bags, but you may find a red bag containing food remains while it is supposed to have dangerous products… You find a black bag with syringes, a piece of the leg [remains of human lower limb]… They [medical personnel] instructed us correctly, but they are not practicing the same… It is very dangerous to us…there are many syringes…many people have been pricked, but they do not disclose…they say ‘I was pricked, but that syringe was burnt… they will never tell you straight* (Incinerator personnel, Region 1)

In the dental unit, the participants perceived themselves at risk following carelessness in handling dental instruments. Also, they felt at risk when treating patients of unknown HIV status. The increased perception of risk of exposure to infection among dental personnel was due to complex invasive procedures the dentists perform to patients. One HCW said:

*We give injections through the mouth; it may happen that you have not followed the principles; there are principles to follow though some of them are very difficult. You may be injecting the dose, and the patient bites you, the blood mixes, mixed blood, and fluids… there are many ways you can be infected; surgery in theatre is one…* (Dental In-charge, Region 2)

Another HCW from a dental related department added to the complexity of dental procedures:

*According to the nature of our duties, the risk is high. As surgeons, we perform surgery. That means we have to give injections; we insert something to remove the tooth or perform minor surgery using a surgical blade. We use the blade… elevator…when the elevator slips away it will tear up the gloves… we are in contact with body fluids every day…the leading risk is sprayed from water and saliva followed by needle prick* (Dental In-charge, Region 1)

Similarly, HCWs from the obstetric ward perceived themselves at higher risk of contracting HIV infection than other units. They felt they were at increased risk because of their regular handling of sharps and blood in the process of delivery of babies:

*To a large extent, we come in contact with blood; during the delivery process we touch blood; you may touch blood from an HIV infected woman… moreover, we know that HIV is transmitted through blood contact…on the other hand, you may prick yourself and acquire infection* (Obstetric ward, Region 2)

The laundry personnel stated that the linens are often stained with blood, and the cleaners may not be sure of diseases the stains carry. They emphasised that the most dangerous time is during the unfolding of linen for the decontami-nation process. Although they could not mention the number of workers who might have been infected through blood stains, they perceived themselves at risk as described below:

*The clothes [linen] are dangerous; the clothes are often stained with blood and pus; I mean a mixture of dangerous contaminants…* (Laundry, Region 2)

The laboratory workers observed that accidents occur in the laboratories. However, they hardly observed any worker who had been exposed to HIV due to such accidents, and all of them knew where to report. One HCW recalled various sources of accidents as follows:

*From sputum, you may acquire AFB [Acid Fast Bacilli], Hepatitis B; you may acquire infections through touching the products…blood, needle prick, body fluids. Even when you collect a specimen, for processing, or disposing of the wastes, if you are not careful you may acquire infections* (Laboratory technician, Region 1)

The HCWs from the Outpatient department were concerned about the handling of casualties. They perceived that they were more at risk in the sense that they receive many casualties with unknown HIV status from road accidents.

The HCWs from units such as mortuary stated that although they were also exposed to various sources of infections, including HIV, they did not have access to adequate protective gears. Therefore, they were worried about their health:

*I can acquire various infections, not only HIV. I can even get yellow fever if I do not have necessary protective gears. If I have bruises and touch the dead body, I can get in contact with body fluids; I may end up with problems such as yellow fever or HIV* (Mortuary, Region 1)

Another HCW added that unlike dead bodies from the ward, it was not easy to predict the type of infection they were being exposed to when they handled dead bodies from outside of which the cause of death was often unknown. They suspected airborne infection from such bodies may spread to healthy people as narrated by one of the HCWs:

*If it [dead body] arrives from the ward, you will know the cause of death because the doctors write the cause on a piece of paper that the death was due to a particular disease. However, if the bodies arrive from outside, you will not know if this one has HIV or not… they bring in dead bodies… air can transmit infection…you cannot leave the body unattended; you have to* (Mortuary, Region 2)

### Availability and Access to Post Exposure Prophylaxis

Most of the HCWs were aware of the reporting chain after exposure to accidents that could put them at risk of HIV transmission. The HCWs from the medical units stated that they mostly reported when they had accidentally pricked themselves, and they got immediate care after reporting. They said that the first step was to report to the In-charge; this facilitated HIV screening procedures for both HCW and the patient, and within 72 hours PEP was initiated depending on the HIV status of the patient. Whilst some of the In-charges were not aware of available records of exposed HCWs or the required PEP dosage, they knew which steps to take immediately after exposure. Several HCWs declared that they attended seminars on Infection Prevention Control (IPC) at least once, and they appreciated the availability of PEP at the workplace:

*Having this programme [PEP] is very useful; it helps people in the sense that immediately when they report the exposure, they access the programme [PEP], and this prevent them from infection* (Medical In-charge –Region Hospital 2)

The HCWs stated that not all of them were able to access the records about injuries at the hospital because these records were often kept in the matron's office and at Care and Treatment Clinics (CTCs). The CTCs were keeping records to monitor the use of PEP. However, PEP was always available:

*We provide PEP to those who were pricked or punched by needles used by AIDS patients… they complete the dose; however, they complain that the dose [PEP] cause discomfort … We advise them to adhere to the dose though* (Medical In-charge, Region Hospital 2)

The HCWs stated that PEP service was made available in 24 hours, and all responsible people were trained:

*The office of supervision is open 24 hours; we believe all managers are conversant with IPC [Infection Prevention and Control] and knowledgeable about PEP. When they receive a person immediately they take precaution; they consult the respective care providers…counselors can provide PEP if needed* (PEP person, Region Hospital 2)

The care and treatment clinics were recognised for conducting refresher courses to enhance knowledge among HCWs. However, it was noted that the majority of HCWs would prefer training outside the workplace.

The Voluntary Counseling and Testing team were not aware of records of people who pricked themselves; however, they stated that they were fully prepared to assist those who pricked themselves. They envisioned the challenge from those who do not disclose after the accidents. The Health secretary in Region 1 stated that she/he had never received a report of a HCW who was injured, but they believed that people were aware of the management of work-related accidents.

Other HCWs reported that they were aware of people who pricked themselves and used PEP, but they never follow them up.

*We had never followed them up. It depends, everybody fears of this. You may stigmatise yourself before being stigmatised* (Assistant nurse In-charge, Region 1)

Almost all HCWs demonstrated an understanding of the reporting channels; the procedure to follow after risky exposure to HIV:

*When the HCW come across any sort of accident, the first step is to report to in-charge… he/she will be tested if he/she had already been infected with HIV; the patient is tested for HIV if infected. After that, if the HCW is uninfected, but the patient is infected, PEP will be given within 72 hours of exposure* (In-charge Medical, Region 1)

On the contrary, some HCWs said that information sharing was limited to certain cadres where some cadres were completely neglected. Mortuary personnel said:

*If you talk to a medical officer or sister In-charge about the safety of mortuary or waste management person, they do not give you a priority compared to nurses or doctors. They [nurses] will tell you that the doctor cannot work without gloves, you see, and he cannot work without a clinical coat, but the mortuary person can even put on a short…* (Waste management, Region 2)

### Motivations and Difficulties of Using Post Exposure Prophylaxis

The HCWs thought that education before exposure to risks was a key motivation to use PEP among HCWs. They stated that health education on risk exposure and available prevention methods among newly employed HCWs would be very useful. They noted that knowing the danger and outcome of mishandling wastes may motivate staff to report immediately when they come in contact with sharps. In most cases, they said they reported, and those who were involved in accidents got appropriate services. One HCW said:

*One staff was injured when he was taking wastes to the damp [incinerator]. Although he wore boots, he was pierced by a needle and reported. Another one was pierced by a needle while maintaining the drainage system, and immediately he reported … I am thankful because they got the services they required* (Waste management, Region 2)

Most of the HCWs voiced the advantages of using PEP:

*Nowadays, there is PEP; it is a medicine which is given within 72 hours. The victim starts it after counseling …it is available all the time…if there is an emergency. Always there is somebody to take care* (Surgical ward, Region 1)

The HCWs knew few colleagues who pricked or cut themselves while handling wastes, and they were able to access PEP and tested negative. They said that once they pricked, they reported to the supervisor who directed them to the counselor; the counselor tested them and gave them PEP. Given the side effects of PEP, users were excused from duties for 3 days. After treatment, they repeated the test; often, they tested negative, and they became role models for teaching others what to do in case of similar exposure.

In most cases, the participants appreciated the outcome of PEP as they witnessed those who were injured and treated:

*It [PEP] works very well…one of my doctors was exposed to HIV when performing his duty and received PEP. I know it helped him. Because of the training he received, when he was exposed, he reported the incident, and he was treated and luckily he ended up safe* (In-charge Theatre, Region 1)

The HCWs stated that there are various mechanisms in place to ensure that all HCWs are equipped with knowledge and skills to control and prevent infections. Training on Infection Prevention and Control and PEP are provided to various cadres of HCWs. One HCW said:

*We [HCWs] were invited to groups. We were taught. Sometimes they gave leaflets. It became easy to understand there is such kind of service. Initially, people did not know, but after training, they know* (In-charge RCH Region 1)

They added that in other sections, all workers, regardless of the profession from higher to lower levels, were trained on IPC and use of PEP. They ensured that everybody attended a monthly meeting where they were reminded about steps to follow and get the correct treatment. Thus, most participants knew that after exposure, one should get PEP within 72 hours. Counselors were perceived to know the logistics involved in PEP use better than other HCWs.

The HCW responsible for providing PEP services added that although the reporting channel after HIV exposure was known, several HCWs might have been injured, but they did not report:

*Possibly, there are people who have been injured while on duty, but they have not reported…there are rules that if one gets injured, he/she must report to the supervisor; the office operates 24 hours… if patients are tested HIV positive, post-exposure prophylaxis is given to victims, but if the victims are also positive they will be treated* (PEP person, Region 2)

Awareness about PEP dose usage was limited to the HCWs who were not directly involved in administering it:

*I know PEP is being used although I may not know if all people prefer to use it. Other people may just wash and squeeze the blood out. I do not know, but during IPC training, we were told to inform the In-charge in case of accidents…The one who got an accident in my unit used PEP did an investigation after that; after finishing the 3 months dose, he tested negative* (In-charge Theatre, Region 1)

Also, it appeared that some HCWs were not keen on reporting:

*People prick themselves and ignore… negative attitudes [towards reporting] disturbs many people. People are not committed to the stated rules at the workplace* (Dental In-charge, Region 2).

In addition, some HCWs recounted that there was little information available on the intended follow up of those involved or exposed:

*It [information] is not strict; when I say it is not strict, I mean those risks for example pricks are not discussed, followed up whether the victim recovered, or is provided with psychological reassurance/support …You know those medicines [PEP] have complications, but nothing is being given to support the victim… it is painful, I mean you just take medicine alone* (Obstetric ward Regional Hospital 2)

HCWs who took PEP may have been affected socially and psychologically. Even if they were not infected with HIV, they had to take Anti-retroviral drugs (ARVs) and felt isolated. Some of them reported to experience side effects after using PEP:

*Some came to me and complained a lot that the medicines [PEP] exacerbate hunger at the same time they have no extra money to buy food frequently… It is beyond ordinary hunger. These are just expressions that you cannot prove, but as a human being, you feel to do something…I have heard that issue of exacerbated hunger from at least 2 people…* (Medical Officer In-charge, Region 2)

## DISCUSSION

We discuss here the perceived risks of occupational exposure to HIV infection and the workplace interventions to reduce HIV transmission. Perception of sources of risks depended on the hospital section in which the interviewed HCW was stationed. Availability, accessibility, and a well-organised reporting channel at the hospital setting contribute to use of PEP services by HCWs.

The majority of the participants reported to have encountered various exposures that they classified as risky. They feared contracting HIV from a risky exposure, and the way in which they recounted their fears gave a strong impressions that these risky exposures were common in their health-care settings. Elsewhere studies have shown that HCWs believed that they were at risk of contracting HIV while working.^15,25^ In the US, a range of Health-care professionals was exposed to HIV infection of which nurses and the physicians sustained nearly identical frequencies of reported needlestick and sharps injuries while following unsafe practices.^9^ Also a previous study in India showed that nursing students had an exposure to blood or body fluid during their nursing practice. Importantly, the most common type of exposure was exposure of intact skin to blood/body fluids followed by needle prick and cut from sharp instruments.^16^

Nevertheless, the risk of transmission of HIV after exposure to body fluids from an HIV-infected patient remains low.^26^ In the current study, the perceived risk of exposure to HIV infection expressed by those working in infectious units such as waste management unit and mortuary call upon hospital administrators to ensure safety to these particular groups given their limited medical-related knowledge. It is important to have strategies for raising their awareness on various risks of occupational exposures that may lead to HIV infection. Similarly, in Kenya, studies have described how support staff had been injured during environmental cleaning. The same study suggested that some HCWs did not report such incidents because they thought the exposure material was noninfectious, ignorant about the risk posed by the exposures or not aware that they were supposed to report.^27^ A study in the United States found that improper reporting of occupational exposure in 17 medical training institutions was due to lack of time.^28^ Strategies are needed to ensure strict adherence to universal precautions and proper handling of occupational exposure to HIV infection among exposed HCWs.

The introduction of PEP has been a significant step towards ensuring safety measures after accidental exposure to HIV infection int the workplace. The management of a person immediately after significant exposure to HIV contaminated blood or body fluids is critically important in minimising the likelihood of HIV transmission. Thus, the availability and access to PEP services are fundamental to achieve the target of minimising HIV infection among HCWs. However, elsewhere, problems in accessing PEP service at the hospital has been reported among professionals after HIV risk exposure.^15^ In the current study, appreciation expressed by interviewed HCWs suggests an acceptance of PEP service. However, inaccessibility of data describing the actual number of those who have been exposed to risk and used PEP poses a challenge to understand the accessibility and uptake of PEP. While other HCWs believed in immediate reporting in order to benefit from PEP, others appeared hesitant and portrayed a more negative attitude towards reporting, a low knowledge on the existence of PEP services appeared to be partially responsible for this reaction. Additionally, participants cited some of the challenges in taking ART that they felt discouraged exposed individuals from reporting and being prescribed PEP, including a fear of side effects and an insatiable hunger. A fear of side effects has been reported elsewhere.^29^ and Care and treatment clinics should ensure optimal support including adherence counselling to PEP users to maximise adherence. Further research, is essential to follow up social status of PEP users and link those in need to appropriate support.

PEP has been proven effective to minimise transmission of HIV infection, and emphasis should be on initiating PEP within 1 to 2 hours of exposure.^30^ However the issue of underreporting remains a challenge especially where people do not follow appropriate channels to report the incidents of exposure to HIV infection risks. In Ethiopia, 68% of HCWs who had been exposed to risk conditions neither reported exposure nor used PEP.^12^ The reasons for underreporting and reporting but not using PEP vary from individual to institutional factors. In the current study on the individual level a fear of side effects from PEP may hinder people from reporting to negate their need to actually take PEP, further a perceived lack of psychological support may discourage people from reporting. At an institutional level there appears to be a lack of value placed on reporting, illustrated by a lack of knowledge amongst senior HCWs of reporting statistics…. In Ethiopia, the major reasons reported for not using PEP after exposure to HIV infection were lack of awareness of the existence of PEP service/protocols, fear of stigma and discrimination, lack of understanding the value of reporting exposures and lack of support and encouragement to report.^12^

In Central India, educational intervention sessions increased knowledge on the use of PEP and risk of HIV transmission due to needle-stick injury and body fluids splashes.^31^ In North West Ethiopia, several respondents took PEP and completed, but some had failed to complete; the reasons for the discontinuity of taking the PEP was found to be fear of its efficacy and the adverse effects.^32^ Nevertheless, there is evidence that PEP reduces the risk of HIV transmission after needle stick injury by 70%.^33^ In order to address the issue of PEP non-use and noncompliance due to side effects, preventive treatment of adverse events may be necessary to enhance the use and ensure completion of HIV PEP.^34^ The revealed motivation and challenges may be used to develop strategies for enhancing PEP use.

### Limitations

The study has a number of limitations that should be considered when interpreting the results. The study took place in just 2 tertiary health facilities and therefore cannot be generalised beyond the context we studied. However it is likely that similar conditions and therefore experiences will be taking place in other tertiary settings across Tanzania. Data for this paper were collected in 2012 and it is possible that changes have been made since this time through a series of National guidelines for the management of HIV and AIDS in Tanzania. However, this paper provides an important benchmark of the experiences of medical and nonmedical HCWs and can be used to assess changes in such experiences over time. This paper is the first in Tanzania to describe the experiences and risks of occupational exposure to HIV.

Our study provides some evidence to make the following recommendations: All cadres working in the health facilities regardless of their profession should be trained on work-related risks of exposure to HIV and the availability of PEP following risk of exposure to occupational health hazards. The statistics of reported injuries/exposure should be shared among all workers to increase awareness and precautions among them. In turn, this may encourage disclosure among exposed HCWs. And finally all workers should adhere to institutional guidelines such as IPC. The management of health facilities should put in place mechanisms to ensue all employees use IPC guidelines and procedures.

## CONCLUSIONS

This study illustrates some knowledge of risk of occupational exposure to HIV and PEP amongst HCWs in the selected hospitals in Tanzania. However, the risk of exposure to HIV infection among the HCWs at workplace appears common. The study illustrated various perceptions and experiences of exposure to risks of HIV infection and PEP use. Nonmedical cadres perceived themselves at higher risk than medical cadres because of handling improperly disposed sharps which calls upon HCWs from various units to use safety boxes to dispose of sharps. Also, the fact that PEP is available and accessible; all HCWs should be motivated to follow institutional guidelines on PEP services after exposure to any suspicious HIV risky sources.
